# An Exposition of the Nature, Treatment, and Prevention of Continued Fever

**Published:** 1836-01

**Authors:** 


ART. II.—*An Exposition of the Nature, Treatment, and Prevention of Continued Fever.* By HENRY M'CORMAC, M.D.—*London,* 1835. 8vo. pp. 202.

IRELAND has long presented a wide field for the investigation of fever, which her enlightened physicians have not left uncultivated. In the Dublin Hospital Reports, and in the Transactions of the College of Physicians, most able monographs have been published from time to time, containing the observations of those who had adequate opportunities of examining the disease on a large scale. They followed the example set them by Sydenham, of describing the epidemic fevers of separate years, or seasons, and they also gave a more ample detail of individual cases, and tabular arrangement, which afforded their readers opportunities of judging for themselves as to the value of the conclusions. From these enquiries have been gained, further proofs of the truth of many of the most important statements of Sydenham, particularly as to the frequency of local complications in fever, and their varying in different seasons of the year, and in different epidemics, circumstances which should be taken into full consideration in the treatment. Dr. M'Cormac has not followed this plan. He has not confined himself to the particular fever of a given spot, but he has written an essay on Typhus Fever in general, compounded of the experience of himself, and of others in various places, and at different periods. "I have," says Dr. M'C., "endeavoured to elucidate the results of my own experience, and supply its deficiencies by a copious reference to the works of the best writers."

Perhaps in the whole range of medicine no subject could be chosen more difficult to execute in this manner than fever. In the examination of a common inflammation, for instance, such as Iritis, or Pneumonia, the observations of others in all periods, and in all countries, can be advantageously selected to complete the writer's own history of these diseases, as they vary but little in all ages and countries; but the same does not hold good in a disease of which the form, complications, treatment, &c. differ so remarkably in different places, seasons, and years, as the disease in question. The materials at our disposal, although abundant, are not sufficiently precise to be woven into one whole, even if a man competent to such a task could be discovered.

But there is another way by which the writings of others can be turned to good account in the investigation of fever, and that is by comparing carefully one's own observations with those of others, or the observations of one writer with another, in order to duly estimate the value of the conclusions of the several investigations as to causes and treatment. Dr. M'Cormac has neither attempted a solid analysis, nor a careful comparison of the observations of the numerous writers whose works he has quoted, but has assumed that continued fever is one and the same disease in all countries, and at all times, and, in framing a catalogue of symptoms and signs, has introd ced those which he has not observed himself from the books of others. As it is quite impossible to separate the results of Dr. M'Cormac's own experience, so as to give the reader any idea of the varieties of fever which he has at any time observed, we shall merely give a sketch of the plan of the work, and of the views adopted by the author, with an estimate of the manner in which it is accomplished.

Dr. M'Cormac states that he has had ample experience of the disease in three different quarters of the globe, and that for some years past he has been professionally connected with the Belfast Dispensary and Fever Hospital, where he has enjoyed almost every additional opportunity that he could wish "for cultivating a very close acquaintance with continued fever in all its forms." He adopts the opinion which was commonly entertained by the older physicians, and which, although occasionally opposed, is still the one most generally received, that fever is not a local disease, but one affecting the whole system. He arranges the phenomena of continued fever under one common head, which he calls Typhus, and makes no subdivisions indicating its varieties. He supposes that the cause of fever (whatever it may be) acts on the nervous system primarily, and thus the functions of assimilation, secretion, animal heat, absorption, sanguification, the external and internal senses, &c. with which the nervous system is either directly or indirectly concerned, are more or less deranged. The frequent occurrence of inflammation of the brain, lungs, and intestines, during the course of fever, is recognized, and the difficulties of detecting them are more strongly dwelt on than the signs by which they are distinguished; the writer rather looking on the general condition of his patient as furnishing indications for treatment, than on the minute investigation and diagnosis of the particular symptoms of diseased structure. There is a short and clear sketch of the morbid anatomy of fever, condensed apparently from the works of French authors, without any allusion to his own experience. The treatment occupies a considerable portion of the volume, and is by far the best part. There is no novelty, but much judicious advice, both in warnings against routine practice, and in urging a careful consideration of circumstances in every individual case. In simple cases he commences with an active purgative, which is repeated in a milder form as occasion may require throughout the disease; and trusts to cleanliness, starving, weak fluids, and cool air. When he sees a case at the onset, he orders an emetic. If there are symptoms of general excitement, he bleeds according to circumstances; but is by no means an advocate for active depletion. If there are symptoms of cerebral inflammation which do not yield to general or local bleeding, and the strength is giving way, he gives calomel and opium, and rubs in mercurial ointment over the scalp, thighs, and arms, so as to affect the mouth; in thoracic inflammations, in addition to antiphlogistics and counter-irritants, he employs the tartar emetic, dissolving one grain in an ounce of peppermint water, and giving one tablespoonful every half hour. He has known injurious effects follow active purging in these cases, and he employs purgatives cautiously. When pneumonia occurs with great nervous prostration, he recommends moderate venesection with antimonials, and counter-irritation by a blister only kept on until the skin is red, or by a piece of flannel dipped in turpentine, and covered with oiled silk, and retained on the chest until sufficient irritation is produced. If, with symptoms of general excitement, there is pain on pressing the epigastrium, he orders twelve or more leeches to the part, and repeats them, or applies turpentine if the pain continues, unless the patient's strength flags, when he desists from depletion. If with general excitement there is diarrhœa he pursues the antiphlogistic treatment, but if the evacuations continue, and the strength flags, he checks them with opium. Whenever there is constipation he gives purgatives: in obstinate cases one drop of croton oil in an ounce of olive oil produces an easy discharge. The employment of opium when perforation has taken place, according to the valuable suggestion of Dr. Graves, is mentioned, but the principles on which it was recommended are not fully stated. He is cautious in administering purgatives if there is diarrhœa, and when constipation exists with considerable prostration, he uses warm water clysters, or the warmer purgatives. He is no advocate for saline medicines, diaphoretics, and diuretics. The administration of stimuli during the period of prostration is considered at length. "If the pulse is soft and small, though quick, and the tongue moist, although not clean, and the patient labour under considerable debility, wine will generally do good." The effect of wine, however, must be carefully watched, and it must be persevered in or discontinued, according to its effects. The diet of the patient during the disease and convalescence is minutely considered, as well as the general management of the bed-chamber, bed, skin of the patient, &c.

The work concludes with observations on the prevention of epidemic fevers, a branch of medical police of vast importance, particularly in Ireland. The crowded state of the confined habitations of the poor of their large cities, ill-ventilated and insufficiently furnished with drains, and the scarcity of food, amounting to actual famine, from the failure of the potato crops, among the country people, are mentioned as fruitful sources of the worst form of fever. Dr. M'Cormac expresses a wish that more corn could be raised in Ireland, so as to substitute more generally bread for potatoes, in order that the poor should not be exposed to the miseries which necessarily arise from the exclusive employment of an article of diet, which cannot be kept (like corn,) from year to year.

In the execution of this volume Dr. M'Cormac has shown that he is practically acquainted with fever, as well as with the works of the best writers on the disease. In his numerous notes he gives ample evidence of his familiarity with the productions of ancient and modern authors relating to every part of his subject, and in almost every language; and in some of them he shews so much power of stating in a condensed form the opinions of others, that we regret the more that he did not confine to the notes the matter which was not the product of his own observations. He has evidently reflected on the most debateable questions arising out of his subject, and if he fails in throwing any new light on what is obscure, he frequently explains with clearness the sources of difficulty. A radical defect, however, in the whole volume is the want of method every where observable, which impairs the utility of his book, and prevents the reader from doing justice to the author. There is no index, no table of contents, no division into chapters, sections, or of any other kind. From the beginning of the book to the end (with the exception of the preface and introduction,) there is so complete a continuity, that no very clear sentences ever mark the transition from the most distinct portion of the subject to the next. The same absence of method is evident in every page: the writer has great difficulty in restraining himself to the division of the subject which he intends to discuss: the examination of symptoms is occasionally interrupted by reference to treatment, the diagnosis is mixed up with the history of the various attendant inflammations; and general moral reflections, maxims of medical ethics, and rules for medical reasoning, are liberally interspersed throughout; all sufficiently excellent in themselves, but calculated, from their indiscriminate intrusion, to distract the attention of the reader from the main question. Dr. M'Cormac writes with freedom, and sometimes with considerable vigour, but he occasionally sins against taste, by the introduction of unusual words, and in the construction of many of his sentences. Thus, "operating the resolution of pneumonia;" "associations of mere intellection," or a theory being "the act of reasoning on facts," are sentences which sadly disfigure the composition.

In thus stating the impression which a careful perusal of Dr. M'Cormac's work has produced upon us, we would by no means convey an idea unfavorable to his abilities; for he undoubtedly possesses considerable talents and acquirements: we only question whether he has employed them as beneficially as if he had adopted the more laborious and satisfactory way of investigating disease which is adopted by our first pathologists, and had given the results of his own observations, not in the form of unsupported general descriptions, but attested by cases, and investigations after death, carefully analyzed, reflected on, and compared.

